# Lipopolysaccharide-Induced Dephosphorylation of AMPK-Activated Protein Kinase Potentiates Inflammatory Injury *via* Repression of ULK1-Dependent Autophagy

**DOI:** 10.3389/fimmu.2018.01464

**Published:** 2018-06-25

**Authors:** Kerui Fan, Ling Lin, Qing Ai, Jingyuan Wan, Jie Dai, Gang Liu, Li Tang, Yongqiang Yang, Pu Ge, Rong Jiang, Li Zhang

**Affiliations:** ^1^Department of Pathophysiology, Chongqing Medical University, Chongqing, China; ^2^Department of Physiology, Chongqing Medical University, Chongqing, China; ^3^Department of Pharmacology, Chongqing Medical University, Chongqing, China; ^4^Hospital of Chongqing University of Arts and Sciences, Chongqing, China; ^5^Department of Emergency, University-Town Hospital of Chongqing Medical University, Chongqing, China; ^6^Laboratory of Stem Cell and Tissue Engineering, Chongqing Medical University, Chongqing, China

**Keywords:** AMP-activated protein kinase, lipopolysaccharide, autophagy, mammalian target of rapamycin, uncoordinated-51-like kinase 1, inflammation

## Abstract

AMP-activated protein kinase (AMPK) is a crucial metabolic regulator with profound modulatory activities on inflammation. Although the anti-inflammatory benefits of AMPK activators were well documented in experimental studies, the pathological significance of endogenous AMPK in inflammatory disorders largely remains unknown. This study investigated the phosphorylation status of endogenous AMPK and the potential roles of AMPK in mice with lipopolysaccharide (LPS)-induced lethal inflammation. The results indicated that LPS dose-dependently decreased the phosphorylation level of AMPK and its target protein acetyl-CoA carboxylase (ACC). Reactivation of AMPK with the AMPK activator A-769662 suppressed LPS-induced elevation of interleukin 6, alleviated histological abnormalities in lung and improved the survival of LPS-challenged mice. Treatment with A-769662 restored LPS-induced suppression of autophagy, inhibition of autophagy by 3-MA reversed the beneficial effects of A-769662. Treatment with A-769662 suppressed LPS-induced activation of mammalian target of rapamycin (mTOR), co-administration of mTOR activator abolished the beneficial effects of A-769662, and the suppressive effects of A-769662 on uncoordinated-51-like kinase 1 (ULK1) phosphorylation. Inhibition of ULK1 removed the beneficial effects of A-769662. These data indicated that LPS-induced dephosphorylation of AMPK could result in weakened inhibition of mTOR and repression of ULK1-dependent autophagy, which might potentiate the development of LPS-induced inflammatory injury. These data suggest that pharmacological restoration of AMPK activation might be a beneficial approach for the intervention of inflammatory disorders.

## Introduction

The energy sensitive serine/threonine kinase AMP-activated protein kinase (AMPK) is a crucial metabolic regulator plays central roles in the maintenance energy homeostasis (1). AMPK is a heterotrimer composed of α, β, and γ subunits (2). The α subunit is the catalytic subunit, the phosphorylation of the Thr^172^ in the α subunit is a hallmark of AMPK activation (3). The regulatory γ subunit binds AMP/ADP under falling energy status and facilitates the phosphorylation of AMPKα and the activation of AMPK (2). AMPK preserves the level of ATP *via* stimulation of energy-producing pathways and suppression of energy-consuming metabolisms (4).

Inflammation includes a series of highly active molecular responses, which requires intensive energy support (5). Interestingly, there is a growing number of evidence indicates that the energy sensor AMPK is involved in the regulation of inflammation, an energy-intensive response (6). It has been reported that activation of AMPK by pharmacological reagents or molecular approaches suppressed the production of pro-inflammatory mediators and promoted the generation of anti-inflammatory cytokines in lipopolysaccharide (LPS)-stimulated macrophages (7–9). In addition, the anti-inflammatory benefits of the AMPK activators have been observed in animal models with colitis, hepatitis, and myocarditis (10–12). Therefore, AMPK is generally regarded as a negative regulator of inflammation (3, 6).

Severe infection-induced systemic inflammation is one of the most serious inflammatory situations with high mortality (13). The lung is the representative organ suffered from systemic inflammation, which is closely associated with the lethal outcomes (14). Recently, the aberrant activation status of AMPK and its pathological significance have been investigated in animal models with acute hepatitis and chronic obstructive pulmonary disease (15, 16), but the potential roles of endogenous AMPK in LPS-induced lethal inflammation remains unknown. In this study, the phosphorylation status of AMPKα in mice with LPS-induced lethal inflammation was determined. And then, the aberrant status of AMPK was pharmacologically reversed, the degree of inflammatory injury and the downstream molecular mechanisms were investigated.

## Materials and Methods

### Animals

The Balb/c mice (male, 6–8 weeks old, weighing 18–22 g) were purchased from the Laboratory Animal Center of Chongqing Medical University (Chongqing, China). The animals were maintained under controlled temperature of 20–25°C with a 12-h light/12-h dark schedule and given food and water *ad libitum*. All experimental procedures involving animals were reviewed and approved by the Institutional Animal Care and Use Committee of Chongqing Medical University.

### Drugs and Reagents

Lipopolysaccharide (from *Escherichia coli*, 055:B5) was purchased from Sigma-Aldrich (St. Louis, MO, USA). The AMPK activator 6,7-dihydro-4-hydroxy-3 -(2′-hydroxy[1,1′-biphenyl]-4-yl)-6-oxo-thieno[2,3-b]pyridine-5-carbonitrile (A 769662), the autophagy inhibitor 3-methyl-3H-purin-6-amine (3-MA), the mammalian target of rapamycin (mTOR) activator 3-benzyl-5-((2-nitrophenoxy) methyl)-dihydrofuran-2(3H)-one(3-BDO), and the uncoordinated-51-like kinases 1 (ULK1) inhibitor N-[3-[[5-cyclopropyl-2-[(1,2,3,4-tetrahydro-2-methyl-6-isoquinolinyl)amino]-4-pyrimidinyl]amino]propyl]-cyclobutanecarboxamide (MRT68921) were the products from Cayman Chemical (MI, USA). The rabbit anti-mouse α subunit of AMPK, phosphorylated α subunit of AMPK (Thr^172^), β-actin, acetyl-CoA carboxylase (ACC), phosphorylated ACC (Ser^79^), ULK1, phosphorylated ULK1 (Ser^757^), light Chain 3 (LC3), and p62 antibodies were purchased from Cell Signaling Technology (Danvers, MA, USA). The rabbit anti-mouse eukaryotic translation initiation factor 4E (eIF4E)-binding protein 1 (4E-BP1), phosphorylated 4E-BP1 antibodies were purchased from Abcam (Cambridge, UK). The enzyme-linked immunosorbent assay (ELISA) kit for determination of mouse interleukin 6 (IL-6) was the product from NeoBioscience Technology Company (Shenzhen, China). The BCA protein assay kit, HRP-conjugated goat anti-rabbit antibody, and enhanced chemiluminescence (ECL) reagents were purchased from Thermo Fisher Scientific (Rockford, IL, USA).

### Experimental Protocol

Lipopolysaccharide was injected intraperitoneally to induce severe inflammation and acute lung injury. To investigate the phosphorylation status of AMPK, vehicle or various doses of LPS (10 and 20 mg/kg, dissolved in normal saline, *n* = 8 per group) was injected, the mice were sacrificed by decapitation 3 h post LPS challenge, and lung sample was harvested for determination of the phosphorylation level of AMPK and ACC with immunoblot analysis.

To evaluate the potential roles of AMPK in inflammatory injury, the mice were treated with the AMPK activator A-769662 (30 mg/kg, dissolved in DMSO, i.p.) 30 min before LPS (20 mg/kg) exposure. The mice were sacrificed at 3 or 18 h (*n* = 8 per group at each time point). Plasma samples and lung sample were collected for further experiments.

To investigate the mechanisms underlying the potential roles of AMPK in inflammatory injury, the autophagy inhibitor 3-MA (15 mg/kg, dissolved in DMSO, i.p.), the mTOR activator 3BDO (100 mg/kg, dissolved in DMSO, i.p.), or the ULK1 inhibitor MRT68921 (50 mg/kg, dissolved in DMSO, i.p.) was co-administered with the AMPK activator A-769662. The mice were sacrificed at 3 or 18 h (*n* = 8 per group at each time point). Plasma samples and lung sample were collected for further experiments.

### Assessment Score

The aberrant physical appearance of LPS-insulted mice was evaluated with the assessment score method as previously described (17). Briefly, the animals were scored according to the appearance of coat, activity, respiration, and posture. The point of coat: 1, smooth; 2, mild ruffling; 3, significant ruffling. The point of activity: 1, normal; 2, moves slowly without stimulation; 3, moves only with stimulation, 4, minimal movement with stimulation. The point of respiration: 1, normal; 2, labored; 3, irregular. The point of posture: 1, moving or resting normally; 2, huddled. The assessment score is the total sum of the points from the above categories.

### Histological Analysis

The lung specimens of mice were immersion fixed in 10% formaldehyde at room temperature for 48 h, and then the specimens were embedded in paraffin. Serial paraffin sections (4 µm) were prepared and stained with hematoxylin & eosin (H&E) routinely for conventional morphological evaluation under a light microscope (Olympus, Tokyo, Japan). The histopathological alterations of the lung tissue were blindly scored according to the method described previously (18). Briefly, the histological abnormalities were graded on a scale of 0–4 (0, normal; 1, light; 2, moderate; 3, strong; 4, intense) for the following features: congestion, edema, inflammation, and hemorrhage. A total score for all of the four parameters was calculated.

### Enzyme-Linked Immunosorbent Assay

To determine the level of IL-6 of the harvested blood sample, the ELISA kit was used to according to the manufacturer’s instructions (NeoBioscience, China).

### Western Blot Analysis

The total protein was extracted from the lung samples, and the protein concentration was determined using BCA method. The protein extracts were separated using 5–10% sodium dodecyl sulfate-polyacrylamide gel electrophoresis and then were transferred to nitrocellulose membranes. After incubation with the blocking buffer (containing 5% skim milk, 10 mM Tris–HCl, 150 mM NaCl, and 0.1% Tween-20) for 1 h at room temperature, the membranes were incubated with primary antibody against AMPKα, phosphorylated AMPKα (Thr^172^), β-actin, ACC, phosphorylated ACC (Ser^79^), ULK1, phosphorylated ULK1 (Ser^757^), 4E-BP1, phosphorylated 4E-BP1, LC3, or p62 overnight at 4°C, followed by incubation with the HRP-conjugated secondary antibody. And finally, the blots were visualized with an enhanced chemiluminescence system (Thermo Fisher Scientific) under the ChemiDoc Touch Imaging system (Bio-Rad Laboratories).

### Survival Analysis

To evaluate the potential effects of the AMPK activator A769662 on the mortality of LPS-challenged mice, the survival of experimental animals was assessed every 6 h for at least 7 days (*n* = 20). The cumulative survival rate was expressed by the Kaplan–Meier curve.

### Statistical Analyses

Final results from the experiments are presented as the mean ± SD. The quantitative data were compared using the one-way ANOVA, followed by the Tukey’s *post hoc* test. The assessment score was compared using the Kruskal–Wallis test. The survival rate was compared using a Kaplan–Meier curve and a log-rank test. Results were considered statistically significant when the *P* value less than 0.05.

## Results

### LPS-Induced Dephosphorylation of AMPK Potentiated Inflammatory Injury

**Figure 1:**
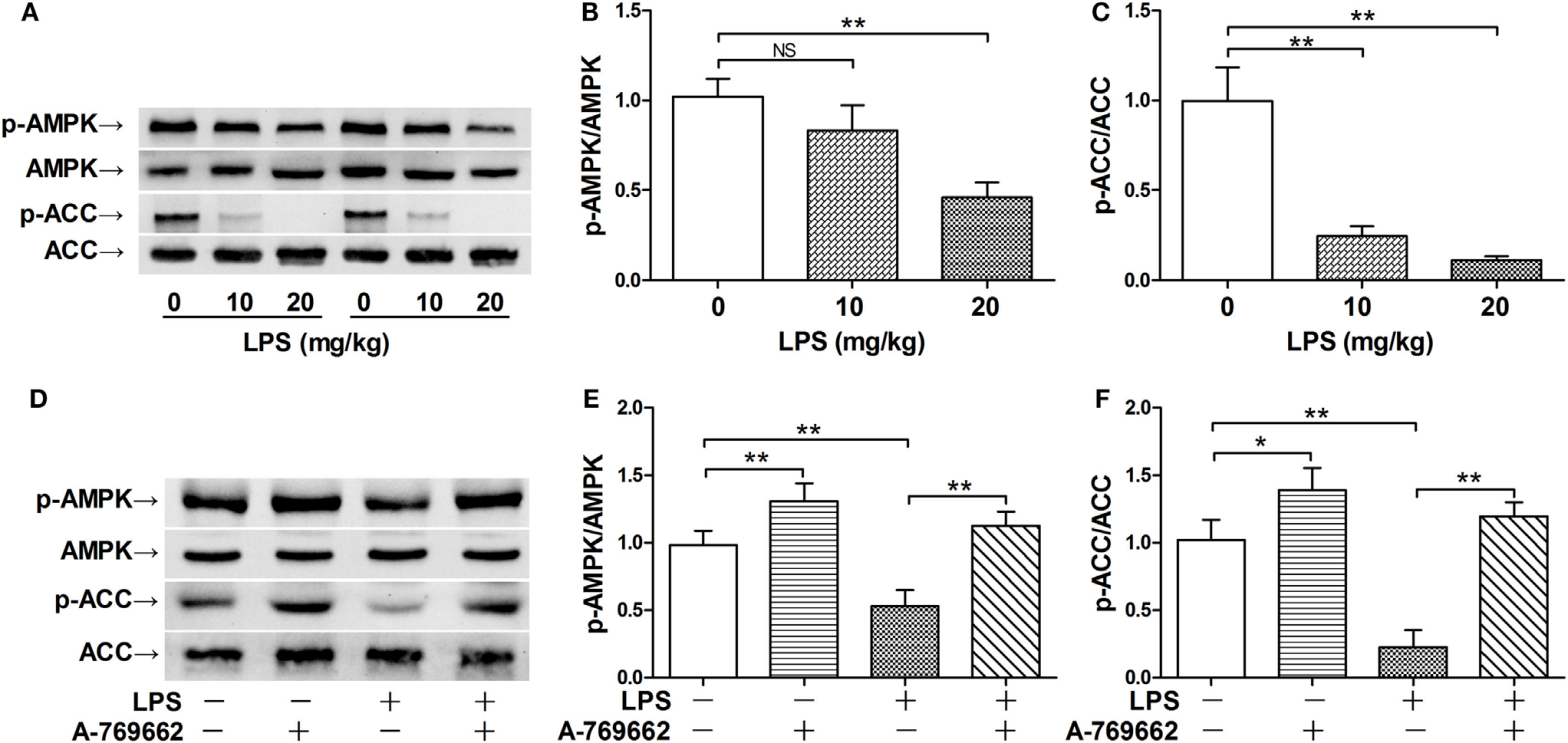
Lipopolysaccharide (LPS) exposure induced dephosphorylation of AMP-activated protein kinase (AMPK). **(A–C)** Mice were exposed to various doses of LPS (0, 10, and 20 mg/kg), lung samples were harvested 3 h post LPS exposure. The level of phosphorylated AMPK (p-AMPK), total AMPK (AMPK), phosphorylated ACC (p-ACC), and total ACC (ACC) was determined by immunoblot **(A)**, and the blots were semi-quantified **(B,C)** (*n* = 4). **(D–F)** Mice were exposed to LPS (20 mg/kg), vehicle or AMPK activator A-769662 was administered 30 min before LPS exposure. The level of p-AMPK, AMPK, p-ACC, and ACC was determined by immunoblot **(D)**, and the blots were semi-quantified **(E,F)** (*n* = 4).

**Figure 2:**
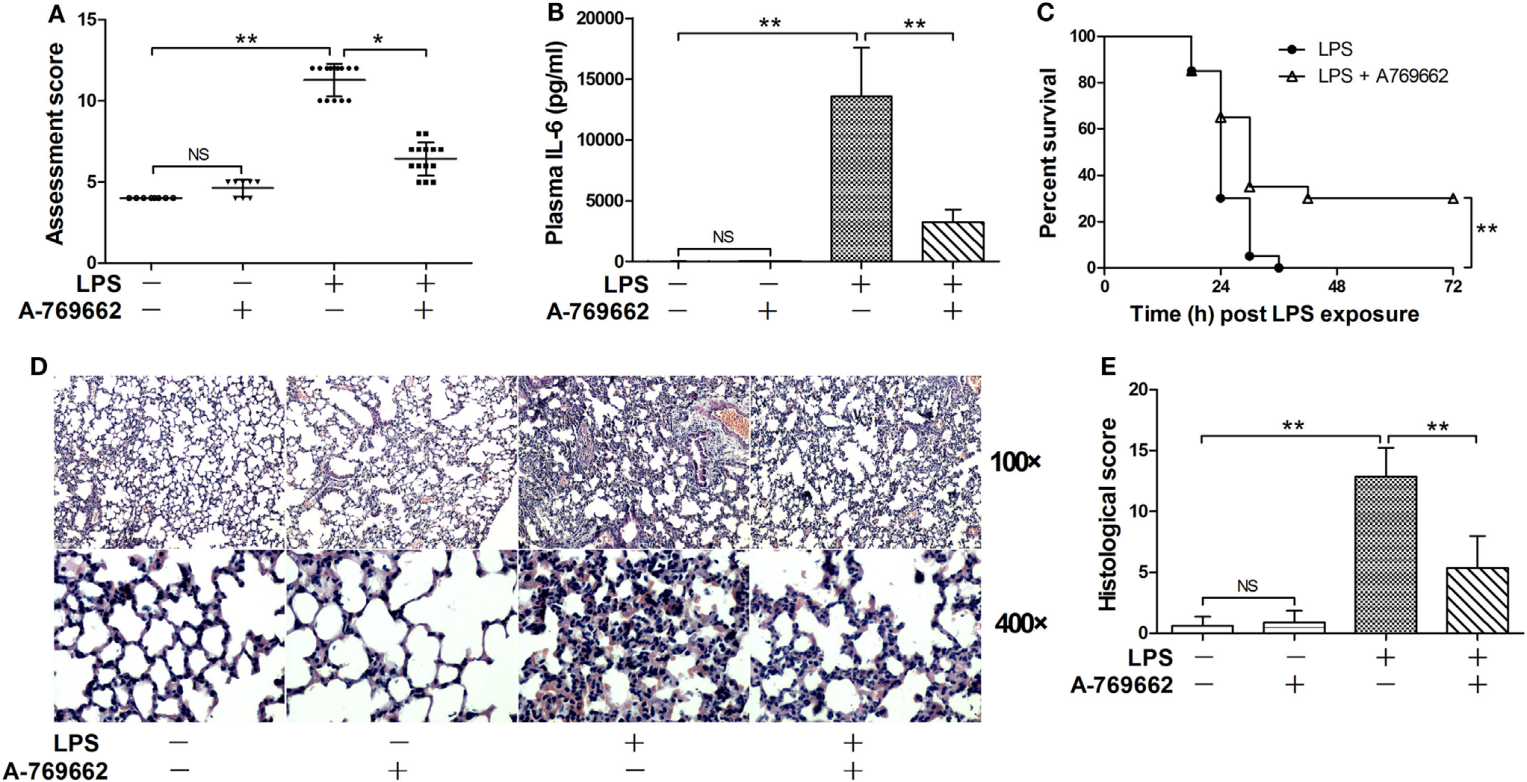
Restoration of AMP-activated protein kinase (AMPK) phosphorylation alleviated lipopolysaccharide (LPS)-induced lethal inflammation. Mice were exposed to LPS (20 mg/kg) with vehicle or AMPK activator A-769662 administration. The physical appearance of the experimental animals was evaluated 18 h post LPS exposure by the assessment scores **(A)** (*n* = 8). The plasma level of interleukin 6 (IL-6) 18 h post LPS exposure was determined by enzyme-linked immunosorbent assay **(B)** (*n* = 8). The survival of the experimental animals was monitored every 6 h for 7 days, and the percent survival rate was expressed by Kaplan–Meier curve **(C)** (*n* = 20). The histological abnormalities of the lung tissue 18 h post LPS exposure were observed with hematoxylin & eosin staining. The representative lung sections of each group were shown **(D)**, the histopathological alterations were blindly scored **(E)**.

The phosphorylation of AMPKα at Thr^172^ is a hallmark of AMPK activation (2). The immunoblot analysis showed that LPS exposure dose-dependently suppressed the phosphorylation of AMPK (Figures 1A–C). Consistently, the phosphorylation of ACC, a representative target of AMPK (19), was also suppressed by LPS (Figures 1A–C). The suppressed phosphorylation of AMPK and ACC could be restored by A-769662 (Figures 1D–F), a widely used AMPK activator (20). In addition, reactivation of AMPK by A-769662 decreased the elevation of assessment score (Figure 2A), suppressed the production of IL-6 (Figure 2B), and improved the survival of LPS-insulted mice (Figure 2C). Meanwhile, LPS-induced histological abnormalities in lung tissue, including alveolar edema, bronchial wall thickening, and leukocyte infiltration, were alleviated by A-769662 (Figures 2D,E). These data suggest that dephosphorylation of AMPK might be involved in the development of LPS-induced inflammation.

### Dephosphorylation of AMPK Was Associated With Impaired Autophagy

**Figure 3:**
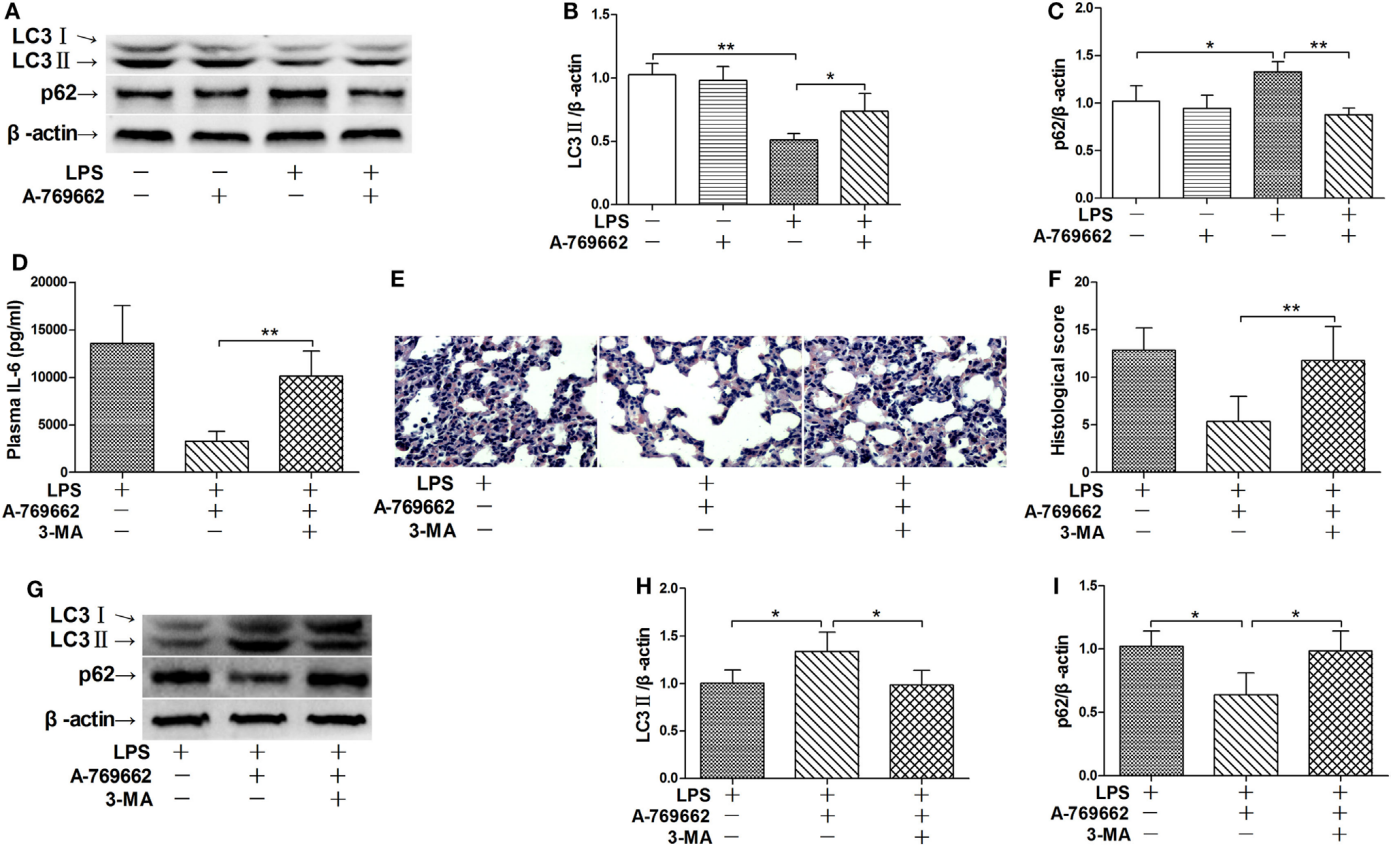
Inhibition of autophagy reversed the beneficial effects of AMP-activated protein kinase (AMPK) activator on inflammatory injury. **(A–C)** Mice were exposed to lipopolysaccharide (LPS) (20 mg/kg) with vehicle or AMPK activator A-769662 administration. The level of LC3II and p62 was determined by immunoblot **(A)**, and the blots were semi-quantified **(B,C)** (*n* = 4). **(D–I)** Mice were exposed to LPS (20 mg/kg) with vehicle, AMPK activator A-769662, and autophagy inhibitor 3-MA administration. The plasma level of interleukin 6 (IL-6) was determined by enzyme-linked immunosorbent assay **(D)** (*n* = 8). The histological abnormalities of the lung tissue were observed with **(H,E)** staining. The representative lung sections of each group were shown **(E)**, the histopathological alterations were blindly scored **(F)**. The level of LC3II and p62 was determined by immunoblot **(G)**, and the blots were semi-quantified **(H,I)** (*n* = 4).

Autophagy was recently suggested to be involved in the progression of inflammation (21). The immunoblot analysis showed that LPS exposure decreased the level of LC3II but increased the level of p62, two molecular markers of autophagy (22), suggesting that LPS suppress autophagy in lung (Figures 3A–C). Reactivation of AMPK by A-769662 reversed the decline of LC3II and the elevation of p62 (Figures 3A–C), suggesting that LPS-induced dephosphorylation of AMPK might result in impaired autophagy. Inhibition of autophagy by 3-MA blocked the suppressive effects of A-769662 on IL-6 induction and histological abnormalities in LPS-exposed mice (Figures 3D–F), these alterations were accompanied with downregulation of LC3II and upregulation of p62 (Figures 3G–I), suggesting that impaired autophagy might contribute to the pathological roles of AMPK dephosphorylation.

### Activation of mTOR Was Relieved by Dephosphorylation of AMPK

**Figure 4:**
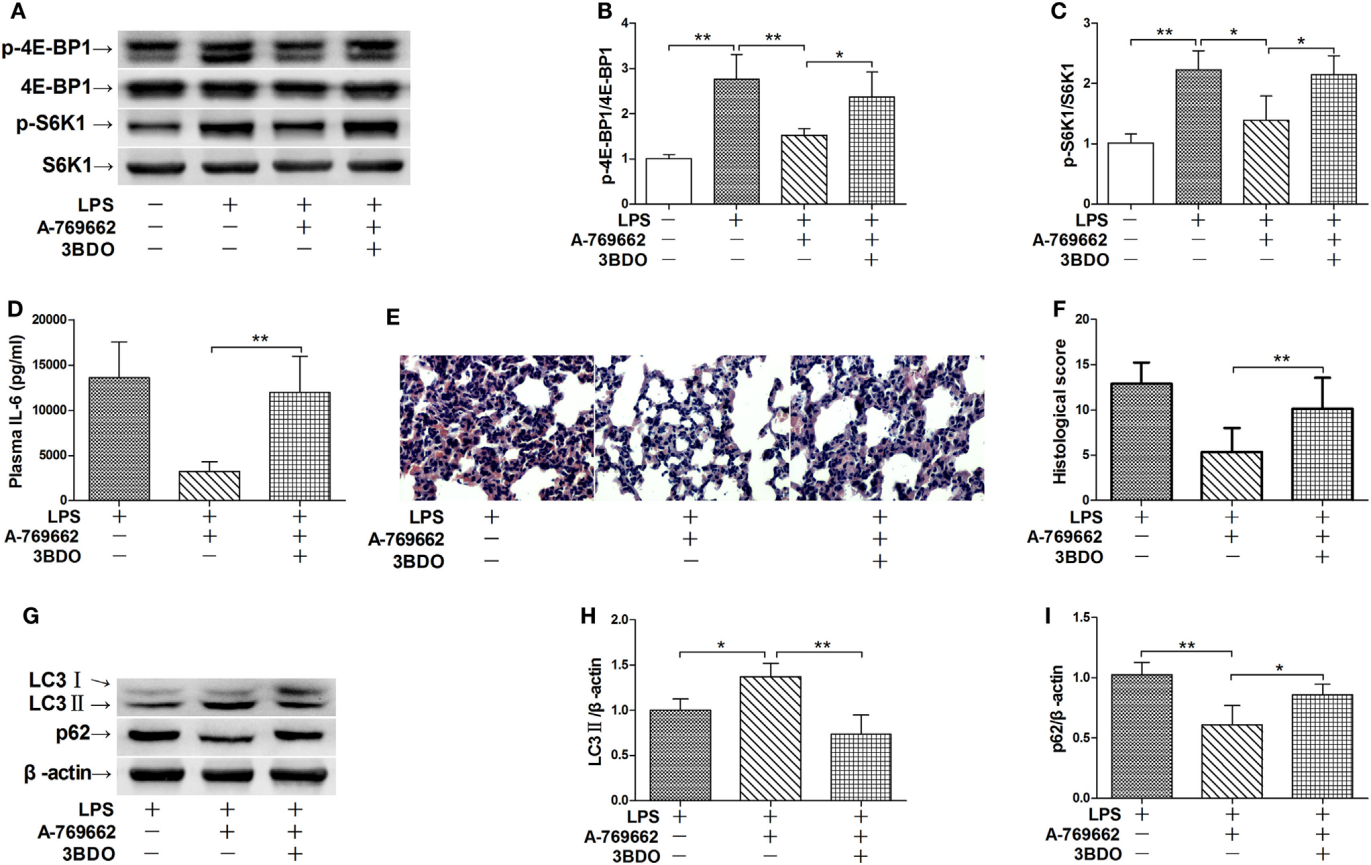
Activation of mammalian target of rapamycin (mTOR) reversed the suppressive effects of AMP-activated protein kinase (AMPK) activator on inflammatory injury and autophgay. Mice were exposed to lipopolysaccharide (LPS) (20 mg/kg) with vehicle, AMPK activator A-769662, and mTOR activator 3BDO administration. The level of phosphorylated 4E-BP1 (p-4E-BP1), total 4E-BP1 (4E-BP1), phosphorylated S6K1 (p-S6K1), and total S6K1 (S6K1) was determined by immunoblot **(A)**, and the blots were semi-quantified **(B,C)** (*n* = 4). The plasma level of interleukin 6 (IL-6) was determined by enzyme-linked immunosorbent assay **(D)** (*n* = 8). The histological abnormalities of the lung tissue were observed with hematoxylin & eosin staining. The representative lung sections of each group were shown **(E)**, the histopathological alterations were blindly scored **(F)**. The level of LC3II and p62 was determined by immunoblot **(G)**, and the blots were semi-quantified **(H,I)** (*n* = 4).

The nutrition sensor mTOR is a crucial regulator of autophagy (23). The immunoblot analysis showed that LPS exposure increased the phosphorylation level of 4E-BP1 and S6K1, two protein targets downstreaming mTOR (24), suggesting that mTOR would be activated by LPS (Figures 4A–C). LPS-induced activation of mTOR were suppressed by the AMPK activator A-769662 (Figures 4A–C), suggesting that AMPK dephosphorylation weaken its suppression on mTOR. Co-administration of 3BDO, an activator of mTOR (25), abolished the suppressive effects of A-769662 on LPS-induced mTOR activation (Figures 4A–C). Activation of mTOR by 3BDO also abolished the negative modulation of A-769662 on IL-6 production and histological lesions (Figures 4D–F). In addition, 3BDO abolished A-769662-induced elevation of LC3II and decline of p62 (Figures 4G–I). These data suggest that AMPK stimulate autophagy *via* inhibition of mTOR.

### ULK1 Mediated the Stimulatory Effects of AMPK Activator on Autophagy

**Figure 5:**
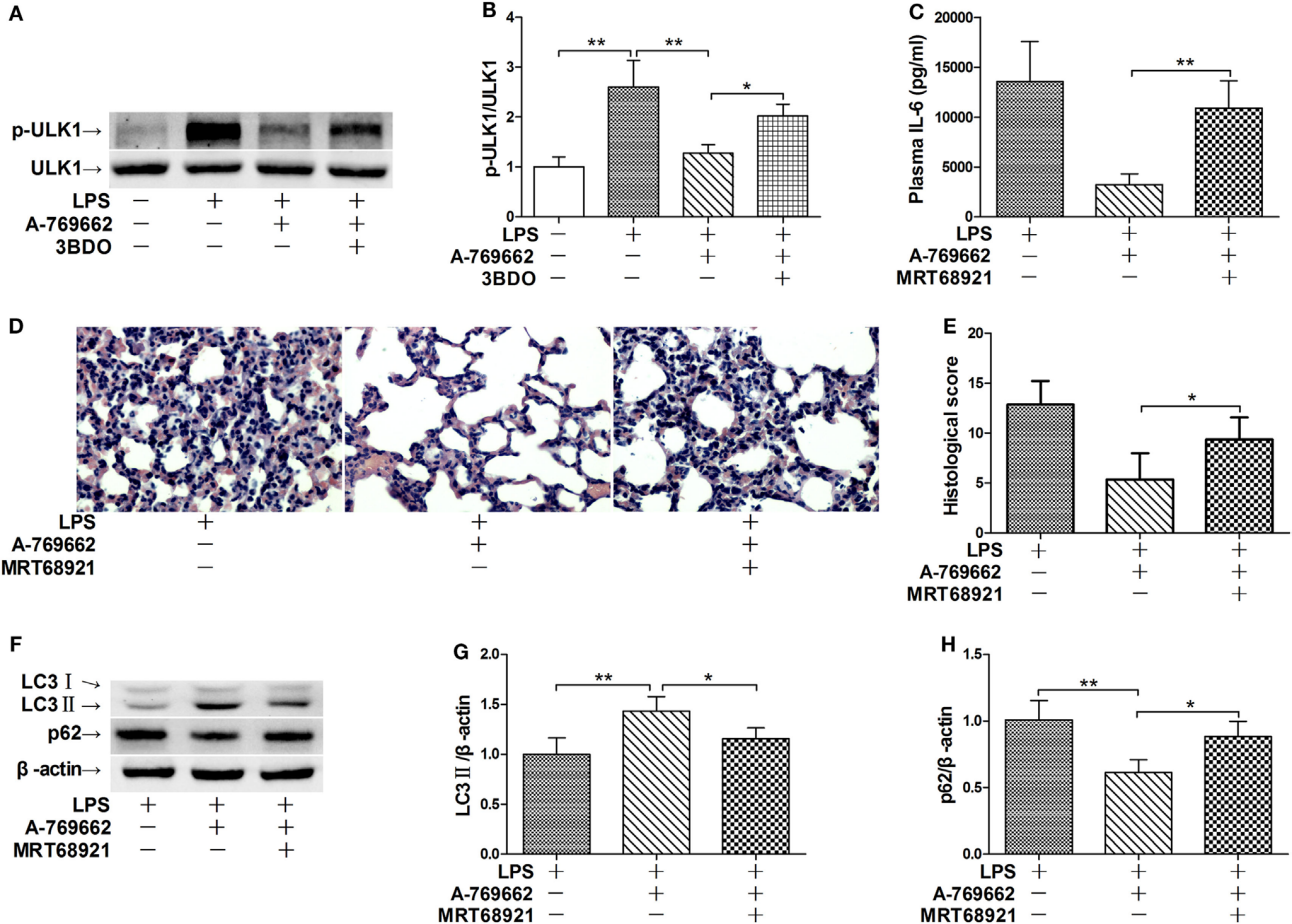
Inhibition of ULK1 reversed the suppressive effects of AMP-activated protein kinase (AMPK) activator on inflammatory injury and autophgay. **(A,B)** Mice were exposed to lipopolysaccharide (LPS) (20 mg/kg) with vehicle, AMPK activator A-769662, and mammalian target of rapamycin activator 3BDO administration. The level of phosphorylated ULK1 (p-ULK1) and total ULK 1 (ULK 1) was determined by immunoblot **(A)**, and the blots were semi-quantified **(B)** (*n* = 4). **(C–G)** Mice were exposed to LPS (20 mg/kg) with vehicle, AMPK activator A-769662, and ULK1 inhibitor MRT68921 administration. The plasma level of interleukin 6 (IL-6) was determined by enzyme-linked immunosorbent assay **(C)** (*n* = 8). The histological abnormalities of the lung tissue were observed with hematoxylin & eosin staining. The representative lung sections of each group were shown **(D)**, the histopathological alterations were blindly scored **(E)**. The level of LC3II and p62 was determined by immunoblot **(F)**, and the blots were semi-quantified **(G,H)** (*n* = 4).

ULK1 is a pivotal initiator of autophagy, which is phosphorylated and suppressed by mTOR (26). The immunoblot analysis showed that LPS exposure increased the phosphorylation of ULK1, treatment with the AMPK activator A-769662 suppressed LPS-induced phosphorylation of ULK1, whereas co-administration of the mTOR activator 3BDO removed the suppressive effects of A-769662 on ULK1 phosphorylation (Figures 5A,B). These data suggest that AMPK suppressed ULK1 phosphorylation *via* repression of mTOR. This study also found that co-administration of MRT68921, an ULK1 inhibitor (27), removed the beneficial effects of A-769662 on IL-6 induction and histological lesions (Figures 5C–E). Co-administration of MRT68921 also reversed the modulatory effects of A-769662 on LC3II and p62 (Figures 5F–H), suggesting that AMPK suppress autophgay and inflammation in an ULK1-dependent manner.

## Discussion

The energy sensor AMPK is a crucial regulator in various energy-intensive physiological and pathophysiological processes (28). The profound anti-inflammatory activities of AMPK have been confirmed both *in vitro* and *in vivo* (6). In this study, we found that LPS exposure induced the decline of AMPK phosphorylation, while restoration of AMPK phosphorylation by the AMPK activator significantly alleviated LPS-induced lethal inflammation. These results suggest that LPS-induced dephosphorylation of AMPK might be a pathological event involved in the development of LPS-induced lethal inflammation, which provides a molecular basis for the pharmacological intervention with the AMPK activators.

The anti-inflammatory properties of AMPK have been well documented (29), but the underlying mechanisms remains largely unknown. It was reported that SIRT1 was an important target mediating the anti-inflammatory effects of AMPK (30, 31). However, recent studies indicated that SIRT1 might be a detrimental factor in LPS-induced lethal inflammation (32, 33). In addition, the metabolic regulatory effects of AMPK are closely associated with mTOR, and activation of AMPK suppresses the activity of mTOR (34). It has been suggested that mTOR play crucial roles in the development of acute lung injury, because pharmacological inhibition or genetic deletion of mTOR resulted in attenuated airway inflammation and lung injury (35–37). Interestingly, several studies have found that inflammatory stimuli activated mTOR both *in vitro* and *in vivo* (36, 38–40). Some studies suggested that LPS-induced activation of PI3K–AKT pathway might be responsible for the activation of mTOR (40, 41). However, this study found that the activation of mTOR might also result from the impaired suppression of mTOR by the dephosphorylation of AMPK in LPS-exposed mice.

There is increasing evidence that mTOR plays pivotal roles in the regulation of inflammation (42, 43), but the underlying mechanisms remain to be investigated. Recently, the crucial roles of autophagy in inflammation regulation have been highlighted (44). In LPS-stimulated human peripheral blood mononuclear cells, inhibition of autophagy resulted in enhanced production of pro-inflammatory cytokines (45). Consistently, macrophages with deletion of autophagy-related 16-like 1 (Atg16L1) also produced higher levels of IL-1β after LPS stimulation and mice lacking Atg16L1 are highly susceptible to dextran sulfate sodium-induced acute colitis (46). The close relationship between AMPK–mTOR and autophagy has been highly concerned (23). Activation of mTOR under amino acid-rich conditions as well as other pathological situations prevents the induction of autophagy (47), whereas inhibition of mTOR by AMPK stimulates autophagy (48). Therefore, suppressed autophagy by dephosphorylation of AMPK and activation of mTOR might be involved in the development of LPS-induced inflammatory injury in this study.

Several mechanisms underlying the inflammation-regulatory effects of authophgy have recently been revealed. It has been reported that autophagy controlled the production of pro-inflammatory cytokines by targeting pro-IL-1β for degradation and by promoting the degradation of pellino E3 ubiquitin protein ligase family member 3 (PELI3), a scaffold protein implicated in LPS-induced activation of pro-inflammatory signaling cascades (49, 50). In addition, increasing NLRP3 degradation and controlling NLRP3 inflammasome activation were also involved in the anti-inflammatory properties of autophagy (51). In mice with LPS-induced inflammatory lung injury or fulminant hepatitis, inhibition of autophagy exacerbated inflammatory injury but activation of autophagy attenuated injury (35, 52, 53). Therefore, restoration of autophagy might be responsible for the beneficial effects of the AMPK activator in this study.

The induction of autophagy is a tightly organized process; ULK1 is the mammalian homologs of yeast Atg1, which plays crucial roles in the initiation of autophagy (54). mTOR is one of the primary negative regulators of autophagy, it suppresses the activity of ULK1, in part, by phosphorylating ULK1 (23, 55). By contrast, AMPK functions as a suppressor of mTOR and an activator of autophagy. AMPK suppressed the activation of mTOR *via* phosphorylating TSC2, an inhibitor of mTOR, and thus prevents the inhibitory phosphorylation of ULK1 and promotes the induction of autophagy (56). Therefore, LPS-induced dephosphorylation of AMPK might result in weakened inhibition of mTOR, which led to enhanced mTOR activation, increased ULK1 phosphorylation, suppressed ULK1 activity, and repressed autophagy.

On the other side, LPS-activated pro-inflammatory signaling significantly modulates the induction of autophagy but the experimental data are highly inconsistent (57). Although LPS induced autophagy in macrophages, endothelial cells, and hepatocytes, it suppressed autophagy in pulmonary epithelial cells (35, 53, 58, 59). Therefore, the modulatory effects of LPS on autophagy seem to be cell dependent. In this study, LPS significantly suppressed autophagy in lung tissue. In agree with our findings, mice with cecal ligation and puncture-induced severe peritonitis also exhibited suppressed autophagy (60). In this study as well as the previously reported study in systemic injury models, the significance of autophagy in certain cells, especially pulmonary epithelial cells, is unclear, which is a major limitation of these studies. In addition, although restoration of autophagy by the AMPK activator improved the total survival rate of LPS-challenged mice, it did not prevent the early death of the experimental animals. The inflammatory status dynamically altered post LPS exposure, which orchestrates the outcomes with other pathological processes such as coagulation and apoptosis (61). The status of autophagy at different stages of inflammatory injury and the significance of autophagy in the regulation of inflammation-related coagulation and apoptosis are worthy of further investigation.

**Figure 6:**
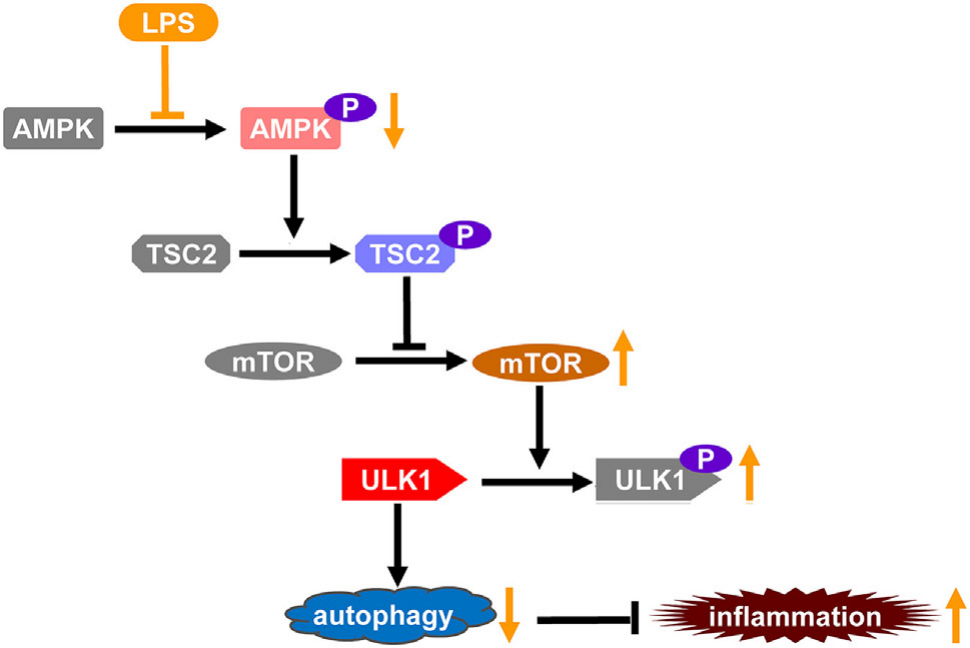
The schematic diagram of the mechanisms through which lipopolysaccharide (LPS)-induced dephosphorylation of AMP-activated protein kinase (AMPK) potentiates inflammatory injury. Autophagy plays crucial roles in the limitation of inflammation, which is a tightly organized process. ULK1 is a key factor promoting the initiation of autophagy, it could be phorphorylated and inhibited by mammalian target of rapamycin (mTOR), a primary negative regulator of autophagy. AMPK functions as a suppressor of mTOR, it suppressed the activation of mTOR *via* phosphorylating TSC2, an inhibitor of mTOR, and thus prevents the inhibitory phosphorylation of ULK1 and promotes the induction of autophagy. LPS exposure induces the dephosphorylation and suppression of AMPK, which might result in weakened inhibition of mTOR, increased inhibitory phosphorylation of ULK1, and repressed autophagy. Thus, LPS-induced dephosphorylation of AMPK and repression of autophagy might be a novel mechanism underlying the development of inflammatory injury.

Taken together, this study found that LPS exposure induced the dephophorylation of AMPK, which play crucial roles in the development of inflammatory injury. The mechanisms underlying the pathological roles of AMPK dephosphorylation include impaired suppression of mTOR and suppressed ULK1-dependent autophagy (Figure 6). Although the underlying mechanisms remain to be further investigated, this study revealed a novel mechanism contributes to the development of LPS-induced acute lung injury.

## Ethics Statement

All experimental procedures involving animals were reviewed and approved by the Institutional Animal Care and Use Committee of Chongqing Medical University.

## Author Contributions

LZ and JW planned the experiments. KF and RJ performed the experiments. LL and QA analyzed the data. JD prepared the figures. YY and PG drafted the manuscript. GL and LZ proofread the final version of the manuscript.

## Conflict of Interest Statement

The authors declare that the research was conducted in the absence of any commercial or financial relationships that could be construed as a potential conflict of interest.
